# Community Engaged Research for Older, Minoritized Americans: Moving the Science of Inclusion Forward

**DOI:** 10.1093/geroni/igaf122.1063

**Published:** 2025-12-31

**Authors:** Tam Perry, Amanda Horn, Vanessa Rorai, JoAnn Smith, James Bridgforth, Jamie Mitchell, Kent Key, Cynthia Howell

**Affiliations:** Wayne State University, Detroit, Michigan, United States; Wayne State University, Detroit, Michigan, United States; Healthier Black Elders Center, Detroit, Michigan, United States; Healthier Black Elders Center, Detroit, Michigan, United States; Healthier Black Elders Center, Detroit, Michigan, United States; University of Michigan, Ann Arbor, Michigan, United States; Michigan State University, East Lansing, Michigan, United States; Flint Healthier Black Elders Center, Flint, Michigan, United States

## Abstract

In celebration of the recent 50th anniversary of the founding of the NIA, the presentation will outline the history and contributions of one of the originally funded Resource Centers on Minority Aging Research and highlights key contributions from the Community Liaison and Recruitment Core of its work in Detroit and Flint, Michigan. Since its inception, this Center has offered community engaged health events, Lunch & Learns, that provide opportunity for older community members to interact with researchers, service providers, and emerging scholars, obtain health screenings from a local university’s mobile health unit and build community among peers. Through these engagement activities where health information is disseminated, trust is fostered as well. The participants are also invited to participate in research studies if interested. This steadfast approach at the program’s Lunch & Learn programs has contributed to sustained attention to best practices for the science of inclusion. This presentation offers a conceptual model of ways to move the science of inclusion forward including values that must be held to be effective and components that will foster the science of inclusion’s possibilities of sustainability. The presentation will end with a discussion of contextual challenges that may affect this conceptual model now and in the future.

